# Evolving Management Paradigms in Dural Arteriovenous Fistulas: From Classification to Personalized Endovascular Therapy

**DOI:** 10.3390/biomedicines13123006

**Published:** 2025-12-08

**Authors:** Veena Shekar, Brandon Lucke-Wold

**Affiliations:** Department of Neurosurgery, University of Florida, Gainesville, FL 32608, USA; veeush2002@gmail.com

**Keywords:** dural arteriovenous fistula, endovascular therapy, cortical venous drainage, venous sinus reconstruction, embolization, precision medicine

## Abstract

Dural arteriovenous fistulas (dAVFs) represent a unique subset of intracranial vascular malformations characterized by pathologic shunting between dural arteries and venous sinuses or cortical veins. Although once considered rare and uniformly high-risk, modern imaging and therapeutic innovations have revealed a spectrum of biological behavior ranging from benign to aggressive. The past decade has witnessed a paradigm shift from purely anatomic classification toward individualized, hemodynamic-based decision-making that incorporates endovascular, microsurgical, and radiosurgical techniques. This Perspective reviews the evolving management of dAVFs, emphasizing early recognition of cortical venous drainage, endovascular innovation, venous sinus reconstruction, and the emerging role of artificial intelligence and personalized medicine in risk stratification. Accordingly, we seek to delineate how a precision approach based on angioarchitecture, patterns of venous flow, and clinical phenotype has transformed the treatment of dAVFs from a purely reactive to a potentially curative discipline.

## 1. Introduction

Dural arteriovenous fistulas are complex lesions with a critical but evolving position in the landscape of cerebrovascular disorders. Once considered to be uncommon curiosities, they now are recognized as potentially curable vascular anomalies. They account for about 10–15% of all intracranial arteriovenous shunts. The acquired arteriovenous connection within the dural leaflets, usually at a venous sinus or cortical vein, represents a defining feature of a dAVF and leads to pathologic arteriovenous shunting, potentially resulting in venous hypertension, ischemia, or hemorrhage [1].

Over the past twenty years, advances in high-resolution digital subtraction angiography, time-resolved MR angiography, and cone-beam CT have substantially improved the ability to detect subtle hemodynamic features and to delineate fistulous anatomy precisely [2]. At the same time, the therapeutic armamentarium has changed, most notably in the domain of endovascular neurosurgery, with liquid embolic agents, balloon-assisted techniques, and transvenous sinus reconstruction having largely revolutionized safety and efficacy.

Despite these advances, DAVFs remain a management challenge, demanding nuanced understanding of the patterns of venous drainage, lesion aggressiveness, and symptomatology. The modern clinician needs to synthesize anatomy, hemodynamics, and patient-specific risk to deliver truly individualized care.

## 2. Classification Systems and Risk Stratification

Historically, classification systems have formed the basis upon which management strategies for dAVF have been developed. The Borden and Cognard classifications remain clinically relevant, each based on the presence and direction of cortical venous drainage [3,4].

**Borden Type I**: Drainage to a dural venous sinus with antegrade flow, minimal risk.

**Borden Type II**: Retrograde flow to cortical veins—intermediate risk.

**Borden Type III**: Direct cortical venous drainage without sinus involvement—highest risk

The classification of Cognard is more detailed, including the direction of venous flow, cortical reflux, and the presence of venous ectasia; this has some predictive value for hemorrhage and neurological deficit.

Yet, modern conception emphasizes that classification needs to be dynamic: some Type I fistulas can indeed evolve into higher-grade lesions through progressive venous outlet obstruction, whereas a few Type II–III lesions remain clinically indolent. Serial imaging and monitoring of symptoms are therefore intrinsic to management.

Emerging radiomics and hemodynamic modeling suggest that venous pressure gradients and outflow impedance may eventually supplant anatomy as predictors of behavior and open avenues to physiologically guided treatment algorithms.

Management of dAVF today requires an understanding of both anatomical and hemodynamic features; a summary of the other existing classification systems will show how they all contribute to decision-making for managing dAVFs. The Djindjian, Lasjaunias, and Lalwani classifications, along with the Cognard classification system, further combine arterial supply patterns, routes of venous drainage, and sinus involvement. Although much more recent literature emphasizes the hemodynamic characteristics such as venous hypertension, timing of reflux, and arterial to venous transit dynamics, these newer concepts are building upon the foundation of the previously established anatomical classification systems, rather than replacing them. By elucidating the relationship between the foundational anatomical classification systems and the newer hemodynamic-driven models, the proposed hemodynamic-driven models can be based upon established terminology rather than being viewed as wholesale replacements for existing classification systems.

## 3. Pathophysiology and Etiology

Most dAVFs are acquired lesions that may develop following venous sinus thrombosis, infection, surgery, or trauma. Chronic venous hypertension promotes angiogenic signaling leading to the formation of pathologic arteriovenous channels within the dural leaflets. Recruited meningeal arteries, typically the middle meningeal, occipital, and ascending pharyngeal branches, maintain fistulous flow [5].

**Figure 1:**
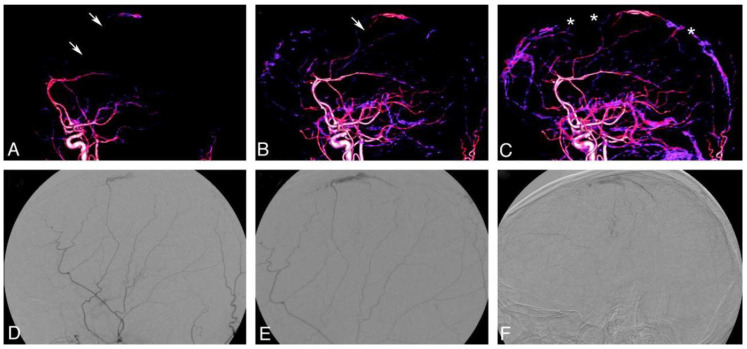
Schematic representation of middle meningeal artery (MMA)–driven neovascularization contributing to dural arteriovenous fistula (dAVF) biology. (**A**) Hypertrophied distal MMA branches supplying the dural leaflets. (**B**) Recruitment of accessory dural arterial feeders. (**C**) Formation of fragile neocapillary plexus along the outer membrane. (**D**) Increased permeability and micro-hemorrhage into the subdural compartment. (**E**) Venous hypertension resulting from impaired venous outflow. (**F**) Integration of these features resulting in a persistent pathologic arteriovenous shunt. Arrowheads indicate the fragile neocapillary plexus that promotes persistent exudation and microhemorrhage. Asterisks (*) indicate filling defects within the superior sagittal sinus consistent with partial venous sinus thrombosis. (Original figure prepared by the authors from Ref. [8]).

Inflammatory mediators including VEGF, bFGF, and MMP-9 have been implicated in the perpetuation of these lesions, suggesting that dAVFs represent a maladaptive wound-healing response rather than congenital malformations [6,7]. This biology has now reframed therapeutic goals beyond the simple disconnection of dAVFs to include the restoration of physiologic venous drainage and the suppression of pro-angiogenic signaling. Angiographic and histopathological studies demonstrate hypertrophy of distal meningeal arterial branches supplying the outer neomembrane and the characteristic “cotton-wool” staining of the neocapillary plexus, which together implicate dural arterial inflow in lesion persistence and recurrence (see Figure 1).

## 4. Clinical Presentation and Diagnostic Challenges

Symptoms of dAVFs range from benign to catastrophic, depending on venous drainage and flow dynamics.

Benign presentations include pulsatile tinnitus, orbital congestion, headache.

Aggressive presentations include intracerebral hemorrhage, seizures, and progressive neurological deficit from venous hypertension.

Diagnoses often rely on high clinical suspicion. MRI findings of engorgement of cortical veins or flow voids raise suspicion, but digital subtraction angiography remains the diagnostic gold standard to identify arterial feeders, venous outflow, and cortical reflux [9].

Increasing emphasis on multiphase CTA and 4D MRA allows for a noninvasive dynamic visualization that will help with the follow-up of previously treated lesions or those that were found incidentally. Artificial intelligence algorithms are in a process of training to automatically flag abnormal flow patterns of venous origin on MR angiograms, which is the next leap toward early detection.

**Figure 2:**
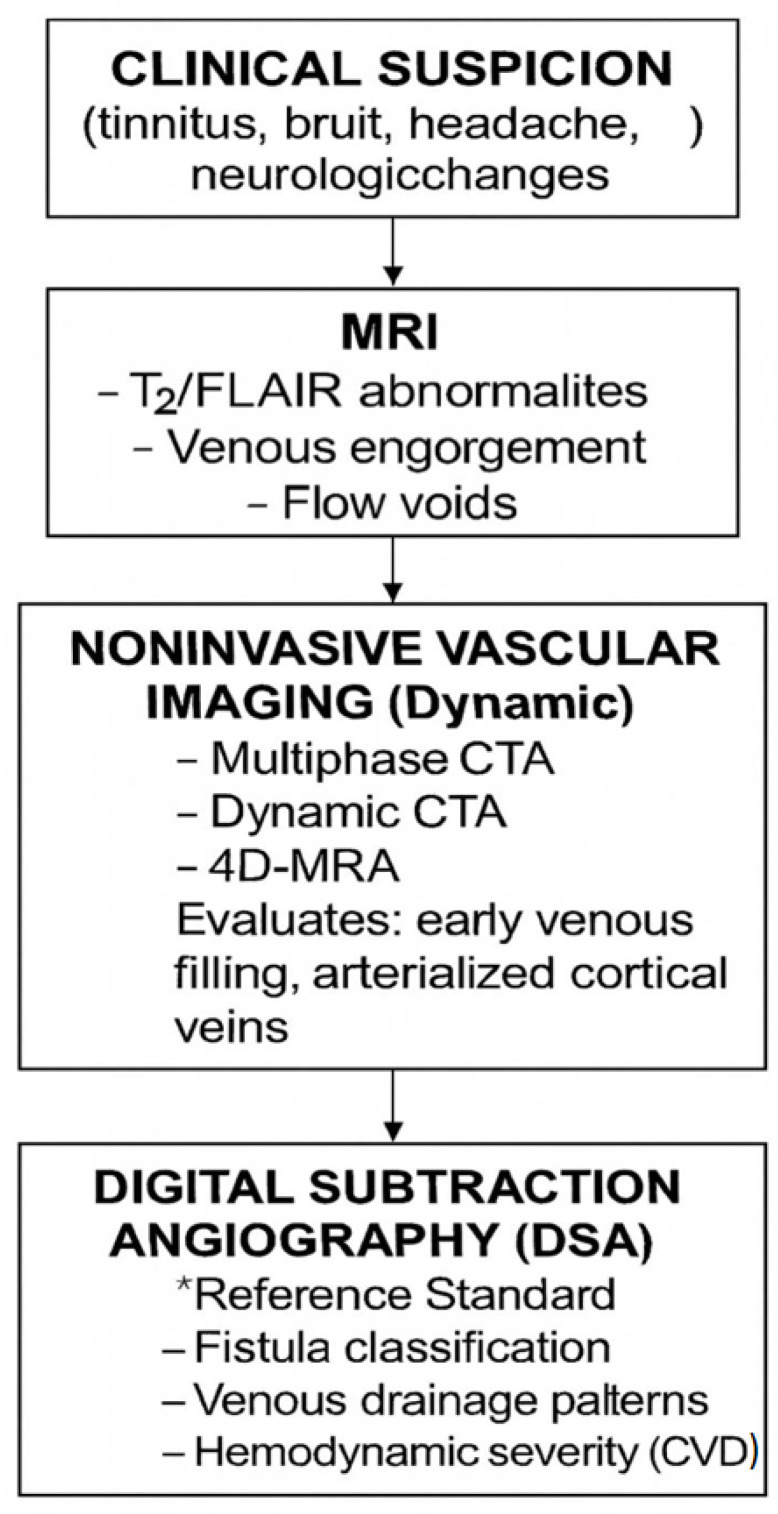
Diagnostic pathway for suspected dural arteriovenous fistula (dAVF). Initial clinical suspicion (symptoms such as pulsatile tinnitus, bruit, or focal deficits) prompts MRI screening to identify venous engorgement or flow voids. Noninvasive dynamic vascular imaging (multiphase CTA/4D-MRA) evaluates early venous filling and arterialized cortical veins; DSA remains the reference standard for definitive classification and hemodynamic mapping. Figure created by the authors.

A practical diagnostic pathway integrates initial MRI screening with dynamic noninvasive vascular imaging (multiphase CTA / 4D-MRA) and culminates in digital subtraction angiography (DSA) for definitive classification and hemodynamic assessment (Figure 2).

## 5. Evolution of Management Strategies

### 5.1. From Surgical Ligation to Endovascular Cure

Historically, dAVFs were treated surgically either by isolating the fistulous sinus or by disconnecting cortical venous drainage. Although effective, morbidity was considerable. Treatment paradigms gradually shifted with the advent of endovascular techniques, initially via transarterial embolization in the 1980s [10].

Liquid embolic agents such as nBCA and, later, Onyx (ethylene-vinyl alcohol copolymer) allowed for deeper and more controlled penetration of the fistulous network. The cohesive, non-adhesive properties of Onyx enabled slower injections in a safer fashion under continuous fluoroscopy, revolutionizing therapy for dAVFs [11].

Transvenous approaches often surpass transarterial routes today, especially for sinus-based lesions. Techniques include sinus packing, partial reconstruction, or focused occlusion of the fistulous pouch and accomplish high cure rates while maintaining physiological venous drainage.

The transvenous embolization of sinus-oriented dAVFs (draining AV fistulas) is the standard technique for treating Cognard IIa–IIb lesions, with stronger evidence supporting your success of long-term occlusion. There are a number of large multi-centre studies indicating 85–95% rates of total occlusion after transvenous sinus packing, exceeding results achieved with transarterial methods of treating sinus-dependent lesions. Transvenous sinus packing has a significant advantage over transarterial methods in terms of the long-term durability of occlusion, fewer re-treatments, and lower rates of recanalization, but only for fistulas draining into the sinus(es), since transarterial or combined methods are better suited for non-sinus (non-sinus) lesions. Once again, it should be emphasized that these findings reflect characteristics inherent in the biology of lesions and demonstrate the need to select embolization techniques based on the specific type of dAVF being treated.

### 5.2. Hybrid and Multimodal Approaches

Multimodal management of complex dAVFs involving multiple feeders or an inaccessible venous outlet relies on the combination of endovascular and microsurgical strategies. Hybrid operating suites allow real-time angiography to be performed during microsurgical disconnection, hence reducing recurrence and improving safety [12].

Radiosurgery plays a role in the treatment of low-flow or residual fistulas that cannot be embolized, with gradual closure over 12–24 months. Careful patient selection seems indicated, however, in view of the risk of delayed hemorrhage during latency.

### 5.3. Reconstruction of the Venous Sinus

Recent innovation has redefined the management of high-grade transverse-sigmoid and superior sagittal sinus dAVFs by restoring rather than sacrificing the sinus. Endovascular sinus reconstruction with balloon angioplasty and stent placement may restore patency, relieve venous hypertension, and induce spontaneous fistula regression. Early results demonstrate durable obliteration while preserving normal venous outflow-an elegant example of physiology-based therapy supplanting destructive intervention.

Reconstructing the venous sinus is a suitable alternative to surgical sacrifice for certain distal arteriovenous fistulae (dAVF) in patients with partial venous sinus thrombosis or stenosis. Several studies of venous sinus angioplasty with stent placement (stenting typically using LVIS, Enterprise or Neuroform stent) have shown increased venous sinus patency, decreased venous hypertension and increased cortical venous outflow. The overall mid-term stenosis rates are reported at approximately 5–15%. Most patients will be managed effectively with dual antiplatelet therapy (aspirin plus clopidogrel) for 3–6 months post operatively. Currently, most of the available evidence regarding venous sinus reconstruction comes from case series or small prospective cohorts; however, the evidence supports the notion that venous sinus reconstruction allows the patient to have the majority of their normal venous drainage pathways intact and completely removes the pathological arteriovenous fistula, thereby providing a durable alternative to transvenous occlusion for maintaining sinus function.

## 6. Decision-Making in the Precision Era

Modern treatment for DAVF is no longer based on simple anatomy but, rather, on individualized hemodynamic profiles and patient contexts. Many decision frameworks now incorporate the following:

- Borden/Cognard Grading of Lesions;
- Symptom severity and venous hypertension;
- Feasibility of access routes and patient co-morbidities;
- Institutional expertise and technological availability.

Overall principle: All lesions with cortical venous drainage or aggressive symptoms should be treated; low-risk lesions may be followed with routine imaging and therapy tailored to minimize morbidity.

For example, machine-learning models have been generated that use angiographic and clinical data to predict fistula aggressiveness and recurrence risk, which therefore enables proactive scheduling of follow-up angiography and intervention.

Multidisciplinary collaboration among neurosurgeons, neuroradiologists, and radiation oncologists offers comprehensive treatment that addresses both the cure and quality of life.

A clinical decision framework for a treatment of dural arteriovenous fistulas (dAVFs) begins with determining the existence of cortical vein drainage. This identifies high risk for complications and an indication for immediate intervention. Subsequent markers of increased vein pressure (such as delayed metabolism; increased number of sided-enlargement of veins; or arterialized brain) add additional guidance to the risk profile of each dAVF lesion in conjunction with mapping the patient’s anatomy. Subsequently, method of access to the venous system (transvenous; transarterial; combined; surgical-hybrid) is considered based on the feasibility of obtaining access to any of the treatments listed. In addition to patient individual factors like age, presence of comorbid conditions (e.g., prescribed anticoagulants) and prior craniotomies, symptoms (tinnitus vs. hemorrhage) must be taken into account. Accordingly, the management options should reflect best practices established from further study of all variables associated with a dAVF lesion. As a result of mapping all variables to an optimal treatment path based on the natural biology of the vascular anomaly, clinicians will have more tools to improve patient outcome with their chosen paradigm of treatment.

## 7. Emerging Trends and Future Directions

The future in the management of dAVFs is defined by innovation at several levels:

1. Next-generation embolic agents: cohesive, radiolucent formulations allowing for deeper penetration while minimizing complications.
2. Microcatheter evolution: Flow-directed and dual-lumen systems that increase control and safety in tortuous anatomy.
3. Artificial Intelligence and Computational Modeling: Automated flow simulations predicting treatment success and recurrence.

In recent years, artificial intelligence (AI) has provided new methods of identifying and understanding dAVFs via their hemodynamics. Using machine-learning techniques based on 4D-MRA, researchers have shown a high degree of sensitivity for detecting abnormal venous filling times and the presence of early cortical venous reflux. Additionally, deep learning systems trained on multiphasic CTA data have been able to identify subtle arterialized cortical veins at a level of accuracy comparable to that of an expert reader. Automated methods of angiographic flow quantification can also assist us in determining arteriovenous transit patterns and studying venous hypertension. While these methods are still undergoing validation, they indicate a move towards increased automation and objective methods for imaging-based phenotyping, risk stratification, and outcome prediction.

1. Transvenous navigation robots and mixed-reality guidance reduce radiation exposure and enhance precision.
2. Molecular targets: Understanding angiogenic signaling may yield adjunct pharmacologic therapies to prevent recurrence after embolization. The ultimate aim is to achieve curative precision with minimal collateral damage-a philosophy embodying the maturation of endovascular neurosurgery.

The traditional focus of treatments has been on providing a technical cure and attaining normal haemodynamics. Nevertheless, functional results of treatment are becoming more apparent, and are at least as important as the technical aspects of treatment. Several reports have shown that by following definitive treatment for a fistula, the modified Rankin Scale (mRS) score improves and the number of neurocognitive symptoms is decreased, particularly in cases where an AVF is causing cortical venous reflux. Furthermore, early treatment of patients who have a fistula may also reduce patient morbidity over time, such as seizures, cognitive decline, or intracranial haemorrhage, resulting in high socioeconomic costs. The integration of patient preferences, quality of life, and cost effectiveness analyses into the treatment plan increases the ability of dAVF management strategies to be translationally relevant and also enhances shared decision making.

## 8. Conclusions

Treatment for dural arteriovenous fistulas has evolved from simple ligation to sophisticated, physiology-driven endovascular care. For the modern neurosurgeon, such lesions are not considered to be purely anatomic but rather dynamic and treatable disorders shaped by individual venous architecture and flow dynamics. As imaging, biomolecular insights, and interventional tools continue to evolve, treatments will increasingly be based on precision rather than generalization, matching therapy not only to the type of fistula but also to its behavior, biology, and patient context. This transition epitomizes a broader evolution within medicine itself—from disease classification to patient-specific intervention. Therefore, the story of dAVF management is not only about technical progress; it is a testament to how thoughtful integration of innovation and empathy can fundamentally redefine outcomes in neurosurgical care.

Management of dural arteriovenous fistulas is transitioning from anatomy-driven paradigms to an integrated model incorporating hemodynamics, venous physiology, and individualized endovascular strategies. As evidence expands for transvenous embolization, sinus reconstruction, multimodal therapy, and emerging AI-guided diagnostics, future practice will likely emphasize precision characterization and tailored intervention. Continued prospective studies and consensus-building efforts are needed to define standardized hemodynamic metrics and optimize patient-centered outcomes.

## Data Availability

Not applicable.
